# The contribution of multiple barriers to reproduction between edaphically divergent lineages in the Amazonian tree *Protium subserratum* (Burseraceae)

**DOI:** 10.1002/ece3.6396

**Published:** 2020-06-17

**Authors:** Tracy M. Misiewicz, Tracey S. Simmons, Paul V. A. Fine

**Affiliations:** ^1^ Department of Integrative Biology, University and Jepson Herbaria University of California Berkeley CA USA; ^2^ Department of Biological Sciences San Jose State University San Jose CA USA; ^3^ Department of Integrative Biology Essig Museum of Entomology University and Jepson Herbaria University of California Berkeley CA USA

**Keywords:** Amazon, postzygotic barrier, prezygotic barrier, reproductive isolation, speciation tropical tree

## Abstract

Disentangling the strength and importance of barriers to reproduction that arise between diverging lineages is central to our understanding of species origin and maintenance. To date, the vast majority of studies investigating the importance of different barriers to reproduction in plants have focused on short‐lived temperate taxa while studies of reproductive isolation in trees and tropical taxa are rare. Here, we systematically examine multiple barriers to reproduction in an Amazonian tree, *Protium subserratum* (Burseraceae) with diverging lineages of soil specialist ecotypes. Using observational, molecular, distributional, and experimental data, we aimed to quantify the contributions of individual prezygotic and postzygotic barriers including ecogeographic isolation, flowering phenology, pollinator assemblage, pollen adhesion, pollen germination, pollen tube growth, seed development, and hybrid fitness to total reproductive isolation between the ecotypes. We were able to identify five potential barriers to reproduction including ecogeographic isolation, phenological differences, differences in pollinator assemblages, differential pollen adhesion, and low levels of hybrid seed development. We demonstrate that ecogeographic isolation is a strong and that a combination of intrinsic and extrinsic prezygotic and postzygotic barriers may be acting to maintain near complete reproductive isolation between edaphically divergent populations of the tropical tree, *P. subserratum*.

## Graphical abstract

Studies of reproductive isolation in both trees and tropical lineages are exceedingly rare. We examined multiple barriers to reproduction in an Amazonian tree species complex, *Protium subserratum* (Burseraceae) which grows parapatrically on white‐sand and non‐white sand soils of the lowland Amazon. Our results suggests that reproductive isolation between lineages is near complete with a combination of intrinsic and extrinsic pre‐ and postzygotic barriers likely to be limiting gene flow between ecotypes.

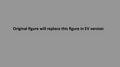

## 1 INTRODUCTION

The origin of reproductive isolation in plants is central to our understanding of the speciation process. Complete reproductive isolation is rarely the consequence of any single isolating mechanism. More commonly, a number of barriers to reproduction will accumulate over time, additively contributing to the total level of reproductive isolation between lineages (Coyne & Orr, 2004). Consequently, assessing the relative importance of many different barriers to reproduction between closely related lineages or species pairs is essential to our understanding of speciation (Coyne, 1992; Coyne & Orr, 2004; Dobzhansky, 1951; Schluter, 2001).

Barriers to reproduction act sequentially to limit gene flow and hence are categorized by the point in an organism's life in which they act. While individual barriers to reproduction may be equally strong in limiting gene flow, early‐acting barriers will have a disproportionately large effect on the level of total reproductive isolation (Coyne & Orr, 2004). In angiosperms, reproductive barriers can be temporally classified into three categories, those that act prior to pollination, those that act after pollination but prior to the fusion of parental gametes (prezygotic barriers), and those that act after the fusion of parental gametes (postzygotic barriers; Grant, 1971). Prepollination, and therefore also prezygotic, barriers to reproduction include geographic and habitat isolation, temporal isolation, and mechanical floral isolation (Grant, 1949; Lowry, Rockwood, & Willis, 2008; Rieseberg & Willis, 2007; Sakaguchi et al., 2017; Schiestl & Schluter, 2009; Widmer, Lexer, & Cozzolino, 2009). Postpollination, prezygotic barriers include competition between conspecific and heterospecific pollen, pollen–pistil incompatibilities and gametic incompatibilities. Finally, barriers to reproduction may be incurred through postpollination, postzygotic isolating mechanisms and include embryo abortion, ecologically based low hybrid fitness, and hybrid sterility (Coyne & Orr, 2004; Dobzhansky, 1937; Mayr, 1942; Rieseberg & Willis, 2007).

To date, studies of reproductive isolation in plants are both taxonomically and geographically limited with the vast majority of studies focusing on short‐lived taxa found in temperate systems (reviewed in Lowry, Modliszewski, Wright, Wu, & Willis, 2008; Lowry, Rockwood, et al., 2008; Baack, Melo, Rieseberg, & Ortiz‐Barrientos, 2015, but see Kay, 2006, Chen, 2013, Johnson, Price, Price, & Stacy, 2015, Hipperson et al., 2016 and Stacy, Paritosh, Johnson, & Price, 2017). Studies of reproductive isolation in trees are exceedingly rare (but see Abadie et al., 2012; Larcombe, Costa e Silva, Tilyard, Gore, & Potts, 2016; Lepais, Roussel, Hubert, Kremer, & Gerber, 2013), and to our knowledge, no study has thoroughly examined reproductive isolating barriers in neotropical tree lineages. Thus, in spite of the striking disparity in tree biodiversity among temperate and tropical biomes, the mechanisms involved in driving and maintaining tropical plant diversity are still poorly understood. Moreover, trees and other long‐lived taxa exhibit significantly different life history characteristics that are likely to impact their short‐ and long‐term evolutionary trajectories. For example, trees typically have very long generation times and thus exhibit high fecundity over many years; they are typically outcrossing and are often capable of dispersing over long distances (Hamrick, Godt, & Sherman‐Broyles, 1992 and Petit & Hampe, 2006). As a result, reproductive isolating barriers in trees may evolve differently from short‐lived taxa.

The genus *Protium* (Burseraceae) is emerging as a model system for understanding the role of adaptation in tropical tree diversification (Fine, Daly, Villa Munoz, Mesones, & Cameron, 2005; Fine et al., 2005; Fine, Zapata, et al., 2013; Fine, Metz, et al., 2013; Fine, Zapata, & Daly, 2014; Misiewicz & Fine, 2014). *Protium* is composed of small to large canopy trees throughout the neotropics with the center of diversity existing in the Amazon where more than 150 of the ca. 200 species are found (Daly, 1987; Daly & Fine, 2018; Fine et al., 2014). Specialization onto different soils is common within the genus and has been particularly well documented on the mosaic of different soil types found across the Western lowland Amazon basin (Fine et al., 2005, 2014). Edaphic specialization onto nutrient poor white‐sand habitat islands as well as common and more fertile clay and brown‐sand soils found in the Peruvian Amazon have occurred independently multiple times within the genus (Fine et al., 2005, 2014).

*Protium subserratum* (Engl.)*,* which is characterized by morphologically and genetically differentiated populations found across different soil types (Daly & Fine, 2011; Fine, Zapata, et al., 2013; Misiewicz & Fine, 2014), provides an ideal system to investigate the importance of multiple barriers to reproduction in a recently diverged lineage. *Protium subserratum* is a soil generalist tree found across the Amazon and has genetically and morphologically differentiated subpopulations endemic to brown‐sand, clay, and white‐sand soils that are often found in parapatry (Daly & Fine, 2011; Fine, Zapata, et al., 2013; Misiewicz & Fine, 2014). *P. subserratum* is included within the section *Papilloprotium*, which is composed of four taxa that include both edaphic specialists and generalists (Daly & Fine, 2011). Vegetative morphological variation has been noted across the range as well as among localized populations (Daly & Fine, 2011). Daly and Fine (2011) grouped *P. subserratum* individuals into four morphotypes based on leaf morphology. Morphotype 1 is restricted to nonwhite‐sand forests in French Guiana, and Morphotype 4 is restricted to Colombia's Caquetá department. Morphotypes 2 and 3 are more widely distributed, with morphotype 2 associated with clay and brown‐sand soils of the central and Western Amazon and morphotype 3 associated with white‐sand soils in the Western Amazon (Daly & Fine, 2011).

Further phylogeographic analysis by Fine, Zapata, et al. (2013) found *P. subserratum* in the Western Amazon formed a well‐supported clade. Within the clade, Peruvian white‐sand individuals, and Brazilian and Peruvian nonwhite‐sand individuals formed nonmonophyletic groupings. Finally, microsatellite analysis of populations, from the Peruvian department of Loreto by Misiewicz and Fine (2014), demonstrated three distinct soil specialist populations, each associated with white‐sand, brown‐sand, or clay soil types. Populations of *P. subserratum* across all three soil types were found to be more genetically similar to geographically distant populations found on the same soil type than to nearby populations found on different soil types. Migration rates were higher between geographically distant populations of the same soil type than between adjacent populations on different soil types, and admixture analyses demonstrated that the presence of adult hybrids among ecotypes is rare. While population genetic analysis suggested the presence of low levels of gene flow across habitat boundaries, populations of both ecotypes clearly maintained their genetic and morphological integrity, suggesting that barriers to reproduction are present (Misiewicz & Fine, 2014).

In this study, we provide a critical missing link in speciation studies by systematically examining multiple barriers to reproduction between diverging lineages of habitat specialist ecotypes of *Protium subserratum* (Burseraceae), an Amazonian rain forest tree. Using an observational approach combined with a hand‐pollination experiments, population genetics, and species distribution modeling we aimed to (a) quantify the contributions of premating barriers to reproduction (ecogeographic isolation, flowering phenology, pollinator assemblage), postmating prezygotic barriers to reproduction (pollen adhesion, pollen germination, and pollen tube growth), and postmating, postzygotic barriers to reproduction (seed development and hybrid fitness) and (b) calculate the total amount of reproductive isolation and the relative contribution of each barrier to total reproductive isolation.

## 2 METHODS

### 2.1 Study system

Soil specialist populations of *P. subserratum* found in the department of Loreto, Peru, typically reach 8–30 m in height and differ from one another in leaf morphology, with individuals found on white‐sand soils exhibiting thick, pubescent leaflets with entire margins, whereas brown‐sand individuals have thinner, glabrous leaflets with some marginal teeth (Fine, Zapata, et al., 2013; Misiewicz & Fine, 2014). Protium *subserratum* is dioecious, and male and female flowers closely resemble each other. Flowers within and between ecotypes are relatively uniform with the exception that the adaxial surface of the petals of the white‐sand ecotype is pubescent (T. M. Misiewicz, personal observation), a character that has been overlooked in previous studies (Daly & Fine, 2011). Flowers are fragrant and nectariferous with small white petals ~5 mm in length. Pollen grains are approximately 6.5 μm in length, invisible to the naked eye. Once a tree begins to flower, it does so abundantly. While the lifespan of an individual flower is about 48 hr, a tree will generally remain at flowering peak for one to two weeks and can produce flowers over one to two months (T. M. Misiewicz, personal observation).

### 2.2 Study populations

**FIGURE 1:**
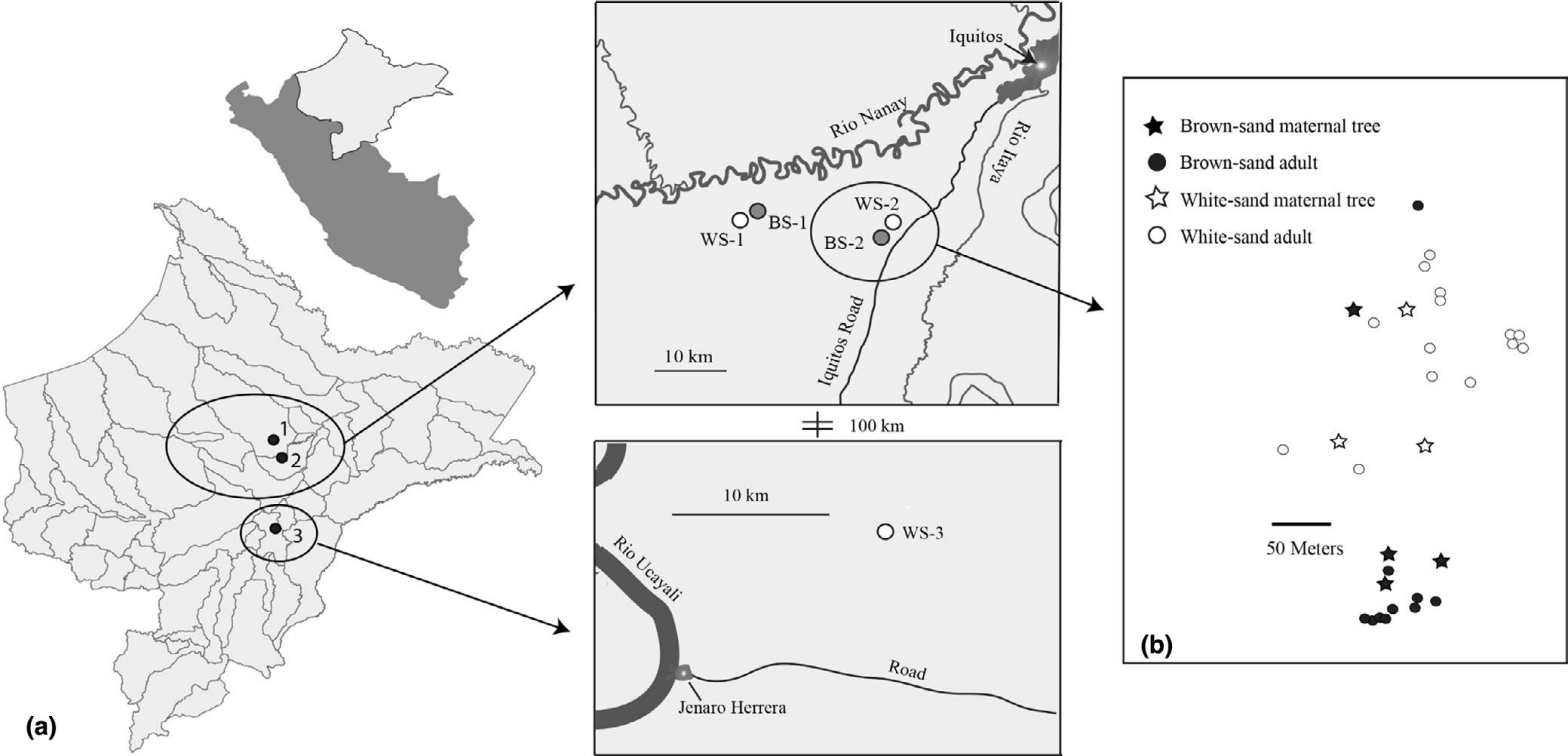
(a) Sample sites and soil types for populations of *P. subserratum* in the region of Loreto, Peru. Numbered points represent the three sites where populations were found. Each individual population used for species distribution modeling (ecogeographic isolation) is displayed in the inset. White circles represent populations found on white‐sand soil, and grey circles represent populations found on brown‐sand soil. All other components of reproductive isolation were investigated in populations BS‐2 and WS‐2. (b) Map of trees found at the contact zone between WS‐2 and BS‐2. Stars indicate maternal trees under which seedlings were collected for genotyping (hybrid fitness)

All components of reproductive isolation were examined using populations of brown‐sand and white‐sand specialist populations in the region of Loreto, Peru (Figure 1, Table S1). Georeferenced locations for white‐sand and brown‐sand individuals from all populations were used for species distribution modeling in order to estimate ecogeograpic isolation (Figure 1a, Table S1). All other barriers to reproduction were investigated using parapatric white‐sand and brown‐sand populations (WS‐2 and BS‐2, Figure 1a) in the Allpahuayo‐Mishana National Reserve in the Amazonian region of Loreto, Peru. Population WS‐2 and BS‐2 are directly adjacent to one another.

### 2.3 Ecogeographic isolation

Ecogeographic isolation occurs when biological differences among taxa influence their geographic distribution (Ramsey, Bradshaw, & Schemske, 2003; Sobel, Chen, Watt, & Schemske, 2010). If suitable habitats are not geographically proximate to one another, taxa occurring in those habitats may experience limited opportunities to interbreed (Sobel et al., 2010). Ecogeographic isolation has traditionally been assessed using common gardens and reciprocal transplants (Baack, 2005; Raabova, Fischer, & Munzbergova, 2008); however, the utility of these methods is limited for rare plant populations or long‐lived taxa such as trees. As a result, niche modeling has been used as an alternative method for understanding the importance of ecological factors in determining geographic distributions of natural populations (Glennon, Rissler, & Church, 2012; Phillips, Anderson, & Schapire, 2006; Sobel, 2014; Sobel & Streisfeld, 2015). We used occurrence data for 180 individuals of *P. subserratum* brown‐sand and white‐sand ecotypes from Loreto, Peru (Figure 1a, Table S1), in combination with SoilGrids250m maps to estimate realized niches for each ecotype and predict the extent to which they overlap.

#### 2.3.1 Soil data

SoilGrids250m provides machine learning based predictions for standard chemical and physical soil properties at seven standard depths and occurrence probabilities distributions for 24 different soil classes (Hengl et al., 2017). Maps for soil types that are not present in the Amazon Basin and those that were highly correlated (0.75 cutoff) were excluded from the study. In total, thirty‐nine soil variables (Table 1) from SoilGrids250m were trimmed to a 90,000 km^2^ area where brown‐sand and white‐sand ecotypes are known to occur and used to create niche models for *P. subserratum* ecotypes.

**TABLE 1:** Strength of individual reproductive barriers calculated for each ecotype

Isolating barriers	White‐sand Ecotype	Brown‐sand Ecotype
Ecogeographic isolation	0.83	0.45
Flowering phenology	−0.23	0.69
Pollinator assemblage	0.82	0.82
Pollen adhesion	—	0.18
Pollen tube germination	—	0.02
Fertilization/seed development	—	0.40

#### 2.3.2 Niche models

**FIGURE 2:**
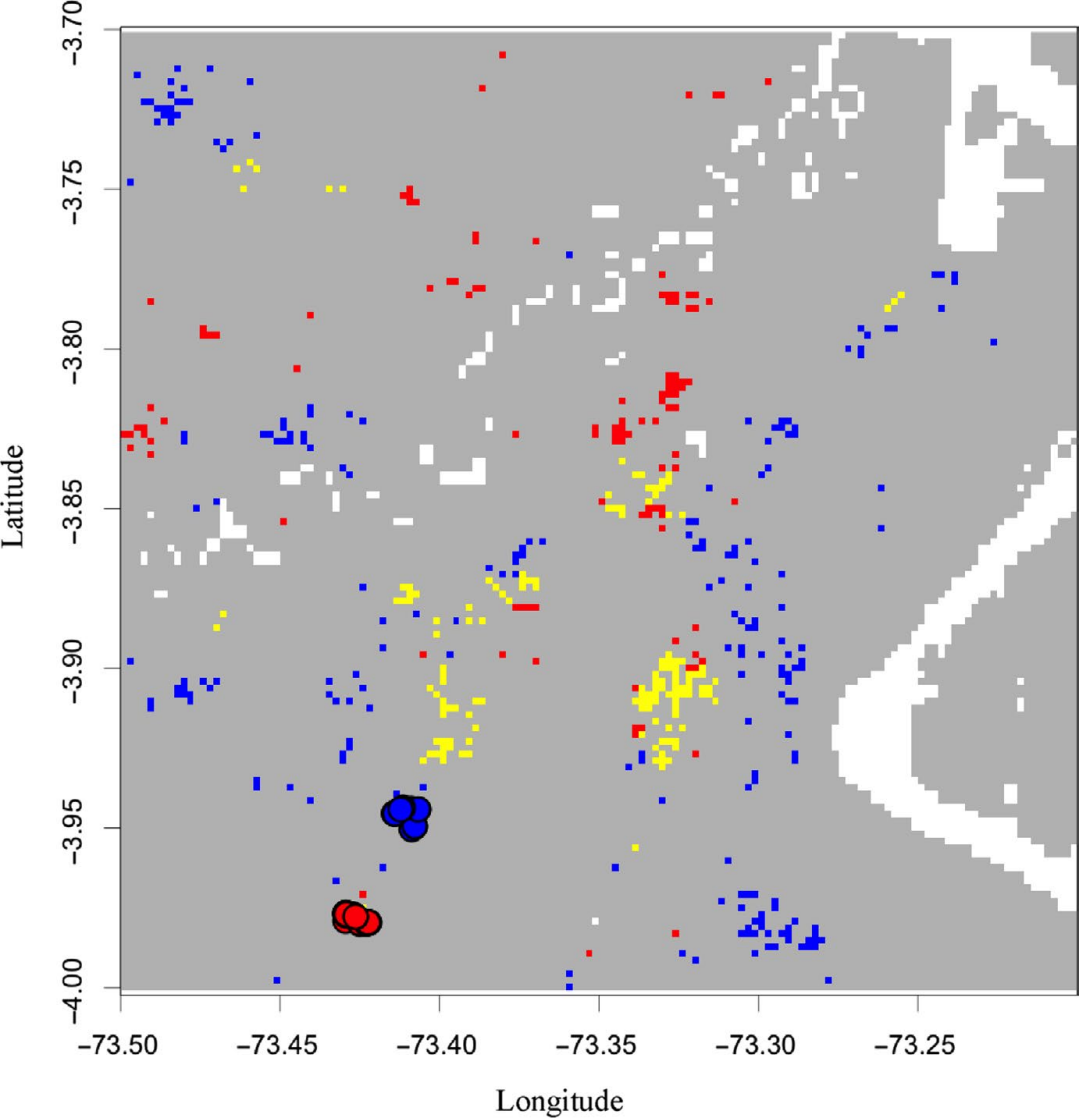
A subset of the total modeled area overlapping species distribution models showing the extent of ecogeographic isolation. Each pixel is 250m x 250m. Habitat predicted by the model to be suitable for white‐sand ecotypes is represented by blue pixels. Habitat predicted to be suitable for brown‐sand ecotypes is represented by red pixels. "Shared" habitat is represented by yellow pixels and includes habitat predicted by distribution models to be suitable for both white‐ and brown‐sand ecotypes and pixels where the pollen dispersal border of one ecotype type overlaps with pixels predicted to be suitable habitat for the other ecotype. Solid dots represent the locations of known white‐sand and brown‐sand individuals within the frame

We predicted the realized niche for white‐sand and brown‐sand ecotypes of *P. subserratum* for a 90,000 km^2^ area in the region of Loreto, Peru, near Iquitos where white‐sand and brown‐sand populations are known to be present. The area was divided into 4.6 million 250 m^2^ grid cells corresponding with the SoilGrids250m soil maps. We used MaxEnt (Phillips, Anderson, Dudík, Schapire, & Blair, 2017) to generate distribution models for each ecotype using SoilGrids250m data and occurrence data for *P. subserratum* brown‐sand (*N* = 76) and white‐sand (*N* = 103) ecotypes. Binary distribution models were generated for both ecotypes by applying an equal training sensitivity and specificity (ETSS) threshold as described in Sobel (2014). Because our habitat modeling took place at such fine spatial scale, we accounted for potential pollen transfer across habitat boundaries by adding a 500 m dispersal boundary around pixels characterized as suitable white‐sand or brown‐sand habitat. Pixels were considered to be shared habitat when there was overlap between the suitable white‐sand and brown‐sand ecotype ranges as predicted by the model or if dispersal boundary pixels for one habitat overlapped with pixels identified as suitable for the other habitat by the model (Figure 2). Differences in soils between white‐sand and brown‐sand ecotype habitats were assessed using a one‐way MANOVA (Wilks *λ*) in *R* (R Core Team, 2018).

### 2.4 Flowering phenology

We quantified the overlap in flowering times by taking observations of brown‐sand (*N* = 39, population BS‐2) and white‐sand trees (*N* = 12, population WS‐2) biweekly from January 2006 to December 2008. Using binoculars, each tree's canopy was surveyed for the presence or absence of flowers. The proportion of individuals flowering in each population was plotted over time.

### 2.5 Pollinator assemblages

Although only relatively minor differences in floral morphology have been observed between *P. subserratum* ecotypes, pollinator preferences for different ecotypes may still exist. For instance, differences in scent or reward may exist between ecotypes. Alternatively, if pollinators themselves are adapted to different habitats, different pollinator communities may be restricted to foraging on geographically proximate plants. In order to determine the extent to which differences in pollinator communities may limit gene flow between ecotypes, we compared pollinator assemblages visiting brown‐sand and white‐sand ecotype trees. Floral visitors to each ecotype were observed using digital video cameras placed in the canopies of six trees: white‐sand and brown‐sand male and female trees (white‐sand male, *N* = 2; white‐sand female, *N* = 2, brown‐sand male, *N* = 1; brown‐sand female, *N* = 1). Video recordings of inflorescences were taken between 0900 hr and 1700 hr between 1 June and 30 July 2012. Each individual recording lasted 15–30 min in duration. A total of 6 hr of video footage (BS = 192 min; WS = 169 min) covering a total of 572 individual flowers were collected (BS, *N* = 313; WS, *N* = 259). All visits were noted, and insects were identified to the lowest taxonomic unit possible. Insect visitors that clearly represented different taxonomic units were further grouped according to morphospecies assignments. A single visit was defined from the time that an individual entered the recording frame to the time it exited. If antagonistic behavior such as nectar robbing was observed, the episode was counted separately and omitted from the final analysis. Data from all male and female white‐sand and brown‐sand ecotype trees were pooled for analysis. We compared pollinator assemblages across ecotypes by comparing visitation to each ecotype for every insect visitor. Differences in visits were tested using Wilcoxon's rank sum tests. To account for differences in the abundance of pollinators visiting each ecotype, we calculated the Bray–Curtis dissimilarity index (Bray & Curtis, 1957; vegdist function; vegan R package; Oksanen et al., 2015). To calculate RI_4C_ (Sobel & Chen, 2014), the Bray–Curtis dissimilarity index value was used for *U* and the value of 1–*U* was used for S (see Calculating Reproductive Isolation).

### 2.6 Postpollination isolating barriers

#### 2.6.1 Hand‐pollination experiments

The following protocol was followed for all hand pollinations described in the sections *Pollen adherence, germination, and pollen tube growth* and *fertilization and seed set*. All trees were climbed by the first author, and female inflorescences were bagged while flowers were still in bud using bags made from synthetic woven interfacing. Male and female inflorescences were checked daily. Upon anthesis of female flowers, we collected newly opened male flowers from neighboring trees for hand‐pollination experiments. Floral bags were removed from female inflorescences, and flowers were hand‐pollinated by gently bringing anthers from the male flower into contact with the receptive stigma of the female flower. Hand‐pollinated flowers were marked with paint on their pedicel, and the inflorescence was rebagged. Inflorescences used in this study supported between 10 and 96 flowers each. No more than 30% of flowers on any given inflorescence were hand‐pollinated, and the remaining flowers were left as negative controls. Inflorescences of flowering female white‐sand trees could not be safely climbed to access their flowers, and as a result, all hand pollinations were made using brown‐sand maternal trees. Only one type of pollination treatment (hybrid or parental cross) was made per inflorescence to avoid selective abortion or preferential resource allocation to some flowers over others.

#### 2.6.2 Pollen adherence, germination, and pollen tube growth

Parental (*N* = 33) and hybrid (*N* = 35) hand pollinations were made using one maternal brown‐sand individual. Hand‐pollinated flowers were collected 48 hr after pollinations were made and fixed for 24 hr at room temperature in 4% paraformaldehyde solution (w/v) in phosphate‐buffered saline (PBS) solution (0.01 M phosphate buffer, 0.0027 M potassium chloride, and 0.137 M sodium chloride). Flowers were then transferred to PBS solution and stored at 20°C. Prior to tissue preparation, petals were removed from the preserved flowers. Floral tissue was cleared and softened in 4 M NaOH for 6 hr, rinsed, and stained with 0.1% decolorized aniline blue (Martin, 1959) for an additional 6 hr and then squashed and mounted in a drop of the aniline blue staining solution for pollen tube visualization. Slides were observed under fluorescence microscopy (Zeiss Axioimager), with UV (400 nm) excitation and photographed (QIClick digital CCD camera). All adhered pollen grains and germinated pollen grains were counted. While initial pollen tube germination was easily observed in all treatments, pollen tubes were not observed growing down the style in either treatment. This result could have been due to a lack of fluorescence in the pollen tubes or because 48 hr was not sufficient for pollen tube growth to extend into the style. The number of adhered pollen grains and proportion of adhered pollen grains showing germination and initial pollen tube growth for each treatment was compared using a Mann–Whitney *U* test.

#### 2.6.3 Fertilization and seed set

A total of 140 hand crosses were made using flowers from brown‐sand maternal trees (*N* = 2) out of which 104 hybrid hand crosses were made with white‐sand males (*N* = 3) and 38 parental crosses were made using brown‐sand males (*N* = 2). Floral bags were removed, and fruit formation was quantified two weeks after pollination once all negative controls were no longer receptive and fruit formation was observable in the hand‐pollinated flowers. An additional 46 inflorescences with a total of 1,166 flowers from the same maternal trees were monitored as positive controls in order to determine the natural pollination rate. Seed set data for parental and hybrid crosses were pooled, respectively, and compared using a Fisher exact test for 2 × 2 contingency table.

#### 2.6.4 Hybrid fitness

##### Sampling and genotyping

Individuals found at the contact zone between the white‐sand and brown‐sand population were used for genotyping (Figure 1b). All adult individuals of *P. subserratum* found were tagged and mapped (white‐sand, *N* = 14; brown‐sand, *N* = 14). Leaves were collected and dried in silica for DNA extraction. A voucher specimen from each population was deposited in the Herbarium Amazonense at the Universidad Nacional de la Amazonía Peruana in Iquitos, Peru (AMAZ), and the University Herbarium at the University of California, Berkeley (UC). Seedlings (*N* = 150) were also collected from the seed shadows of white‐sand (*N* = 3) and brown‐sand (*N* = 3) maternal trees at the contact zone where male and female trees of both ecotypes are found within 10 m of each other to test for the presence of hybrids (see Section 2.7).

All individuals were genotyped using thirteen nuclear microsatellite markers previously developed for *P. subserratum* (Misiewicz, Barbosa, & Fine, 2012) and shown to be effective in population level differentiation and the identification of hybrids between soil ecotypes (Misiewicz & Fine, 2014). DNA extraction and genotyping protocols followed those described in Misiewicz et al. (2012).

##### Genetic variation, Hardy–Weinberg equilibrium, null alleles, and linkage disequilibrium

Number of alleles (*A*), observed and expected heterozygosities (*H*_o_ and *H*_e_), the inbreeding coefficient (*F*_IS_), deviation from Hardy–Weinberg equilibrium (HWE), and linkage disequilibrium (LD) for each population were calculated across all loci as described in Misiewicz and Fine (2014).

#### 2.6.5 Ecotypic differentiation and hybrid assignment

Genetic differentiation among different size classes was assessed using comparisons of *θ* calculated with and without correction for null alleles with FreeNA (Chapuis & Estoup, 2007). Population genetic structure was assessed using STRUCTURE 2.3.3 (Pritchard, Stephens, & Donnelly, 2000) as described in Misiewicz and Fine (2014). The model‐based clustering method of NewHybrid version 1.1b (Anderson & Thompson, 2002) was then used to assign individuals to one of six hybrid classes, white‐sand parental, brown‐sand parental, F1 hybrid, F2 hybrid, white‐sand backcross hybrid, or brown‐sand backcross hybrid using the methods described in Misiewicz and Fine (2014). Individuals assigned to a hybrid class with a posterior probability (pp) >90% were considered to be hybrids between the two ecotypes.

In order to test the prediction that hybrid individuals are less fit than parental type offspring, we compared genotypes of first‐year seedlings, juveniles, and adult trees (age classes defined below). We predicted that if hybrids were less fit than parental types, we would observe a higher relative abundance of F1 hybrids in first‐year seedlings and that they would decrease in frequency as age class increased due to environmental filtering. First‐year seedlings (*N* = 74 white‐sand; *N* = 43 brown‐sand) were identified by their persistent seed coat, no more than two leaves, and a height under 10 cm. Juvenile seedlings (*N* = 19 white‐sand; *N* = 14 brown‐sand) had two or more leaves present with heights ranging from 15 to 30 cm. All seedlings were collected within a 15 m radius of maternal white‐sand (*N* = 3) and maternal brown‐sand (*N* = 4) trees found at the contact zone between the two ecotypes. All known adult trees (*N* = 14 white‐sand; *N* = 14 brown‐sand) from the two populations were also sampled and genotyped. All individuals were genotyped and assigned to hybrid classes as described in Misiewicz and Fine (2014).

### 2.7 Quantifying reproductive isolation

The strength of individual prepollination isolating barriers which are based on spatial occurrence (ecogeographic isolation and pollinator assemblages) was calculated using the RI_4_ equations following Sobel and Chen (2014). Reproductive isolation based on differences in flowering time (RI_flowering time_) and isolation due to differences in pollinator assemblages visiting each ecotype (RI_pollinator assemblage_) were calculated using RI_4C_ (Sobel & Chen, 2014). In this equation, *S* is the proportion of pollinators or time that are shared with the other ecotype and *U* is the unshared proportion. RI values range from 0 to 1 with 0 signifying complete overlap and 1 signifying complete isolation (Sobel & Chen, 2014). RI_4C_ was calculated in both directions.

$${\text{RI}}_{4C}=1-\left(\frac{S}{S+U}\right)$$

RI_temporal_ was calculated using RI_4S2_ from Sobel and Chen (2014), which accounts for the abundance of flowering individuals on the probabilities of conspecific and heterospecific pollen transfer over the temporal series. RI_4S2_ was calculated in both directions. *A_i_* and *B_i_* represent the number of white‐sand and brown‐sand trees flowering during month *i*, and *A*_total_ and *B*_total_ represent the total number of white‐sand and brown‐sand trees monitored. RI values for this equation range from 1 to −1 where 1 represents no gene flow, 0 represents random mating, and −1 represents complete disassortative mating (Sobel & Chen, 2014).

$${\text{RI}}_{4S2}=1-2\left(\frac{\frac{\sum i\left(\frac{A_{i}}{A_{\text{total}}}\times \frac{B_{i}}{A_{i}+B_{i}}\right)}{B_{\text{total}}/\left(A_{\text{total}}+B_{\text{total}}\right)}}{\frac{\Sigma_{i}\left(\frac{A_{i}}{A_{\text{total}}}\times \frac{B_{i}}{A_{i}+B_{i}}\right)}{B_{\text{total}}/\left(A_{\text{total}}+B_{\text{total}}\right)}+\frac{\sum i\left(\frac{A_{i}}{A_{\text{total}}}\times \frac{A_{i}}{A_{i}+B_{i}}\right)}{A_{\text{total}}/\left(A_{\text{total}}+B_{\text{total}}\right)}}\right)$$

The strength of individual postpollination isolating barriers (pollen adherence, pollen tube germination and fertilization and seed set from hand‐pollination experiments) was calculated using the RI_4A_ baseline according to Sobel and Chen (2014) where H represents the number of heterospecific matings and C represents the number of conspecific matings. Similar to RI_4S2,_ values for this equation range from 1 to −1 where 1 represents no gene flow, 0 represents random mating, and −1 represents complete disassortative mating (Sobel & Chen, 2014). Because all maternal trees were brown‐sand ecotypes, RI_4A_ was only calculated in one direction.

$${\text{RI}}_{4A}=1-2\left(\frac{H}{H+C}\right)$$

Total RI was calculated using the equation for RI_4E_ from Sobel and Chen (2014) which accounts for barriers that impact co‐occurrence and assumes that reductions in gene flow due to earlier‐acting barriers will limit the extent to which later‐acting barriers can limit gene flow. We used 0.5 as our probability of gene flow which assumes random mating. In order to better understand which barriers are most important at contact zones, we also calculated the relative and total contribution of each barrier excluding ecogeographic isolation.

$${\text{RI}}_{4E}=1-2\times \left(\frac{S_{\text{total}}\times P\left(H|S\right)+U_{\text{total}}\times P\left(H|U\right)}{S_{\text{total}}\times P\left(H|S\right)+U_{\text{total}}\times P\left(H|U\right)+S_{\text{total}}\times P\left(C|S\right)+U_{\text{total}}\times P\left(C|U\right)}\right)$$

## 3 RESULTS

### 3.1 Prepollination isolating barriers

#### 3.1.1 Ecogeographic isolation

2,690 pixels were identified by our model as habitat suitable for the white‐sand ecotype, 627 pixels were identified by our model as habitat suitable for the brown‐sand ecotype, and 96 pixels were identified by our model to be suitable habitat for both ecotypes and therefore “shared.” Once pollen dispersal distance was taken into account, an additional 408 pixels were reclassified as “shared” resulting in a final count of 2,485 white‐sand only habitat pixels, 424 brown‐sand only habitat pixels, and 504 “shared” pixels which included pixels that provided suitable habitat for both ecotypes or were in close enough proximity that pollen dispersal could reasonably occur across soil types (Figure 2). RI_ecogeography_ was calculated as 0.45 for the brown‐sand ecotype and 0.83 for the white‐sand ecotype (Table 1). MANOVA revealed significant differences in 23 soil variables associated with each ecotype (*p <* .01; Table 2).

**TABLE 2:** Results of MANOVA on soil variables used for niche modeling

Soil variable	*df*	Sum of squares	*F* value	Pr (>*F*)
Probability of occurrence of R horizon	1	545.58	77.54	1.16E−15***
Absolute depth to bedrock (in cm)	1	2,806,654.00	8.87	0.003305**
Soil pH x 10 in KCl	1	13.82	18.04	3.50E−05***
WRB 2006 class, Acric Plinthosols	1	15.35	14.77	0.000168***
WRB 2006 class, Albic Arenosols	1	123.35	68.54	2.94E−14***
WRB 2006 class, Alic Nitisols	1	28.50	22.58	4.16E−06***
WRB 2006 class, Ferralic Arenosols	1	1.89	1.10	0.2955
WRB 2006 class, Fibric Histosols	1	3,770.50	50.35	2.98E−11***
WRB 2006 class, Haplic Acrisols	1	496.16	36.20	9.97E−09***
WRB 2006 class, Haplic Acrisols Ferric	1	0.16	0.88	0.3491
WRB 2006 class, Haplic Acrisols Humic	1	0.00	0.74	0.3919
WRB 2006 class, Haplic Alisols	1	585.55	52.66	1.20E−11***
WRB 2006 class, Haplic Arenosols	1	0.11	0.12	0.7308
WRB 2006 class, Haptic Ferralsols Xanthic	1	3.38	26.91	5.80E−07***
WRB 2006 class, Haplic Fluvisols Dytric	1	20.86	14.77	0.000169***
WRB 2006 class, Haplic Gleysols Eutric	1	0.76	15.43	0.000122***
WRB 2006 class, Haplic Lixisols	1	111.53	31.03	9.30E−08***
WRB 2006 class, Haplic Lixisols Chromic	1	0.04	2.25	0.135
WRB 2006 class, Haplic Luvisols	1	0.34	2.04	0.1546
WRB 2006 class, Haplic Nitisols Rhodic	1	43.03	39.65	2.33E−09***
WRB 2006 class, Haplic Planosols Dystric	1	0.00	0.74	0.3919
WRB 2006 class, Haplic Planosols Eutric	1	1.10	2.30	0.1315
WRB 2006 class, Haplic podzols	1	30.58	15.19	0.000138***
WRB 2006 class, Hypoluvic aerosols	1	0.92	17.24	5.12E−05***
WRB 2006 class, Lithic Leptosols	1	0.37	10.33	0.001554**
WRB 2006 class, Plinthic Acrisols	1	2.47	15.00	0.000151***
WRB 2006 class, Umbric Ferralsols	1	0.15	3.43	0.06556
WRB 2006 class, Vertic Cambisols	1	3.51	34.49	2.07E−08***
USDA 2014 class, Aquents	1	0.05	0.74	0.3912
USDA 2014 class, Aquults	1	0.00	0.00	0.9737
USDA 2014 class, Orthods	1	0.01	0.02	0.8989
USDA 2014 class, Rendolls	1	0.00	0.74	0.3919
WRB 2014 class, Udalfs	1	8.48	46.37	1.47E−10***
USDA 2014 class, Udults	1	823.60	10.09	0.001763**
USDA 2014 class, Ustox	1	7.51	1.25	0.2647
Texture class (USDA system)	1	5.24	9.77	0.002069*
Available soil water capacity (volumetric fraction) until wilting point	1	223.53	20.86	9.23E−06***

*** *p* = 0..

** *p* < .001.

* *p* < .01.

#### 3.1.2 Flowering phenology

**FIGURE 3:**
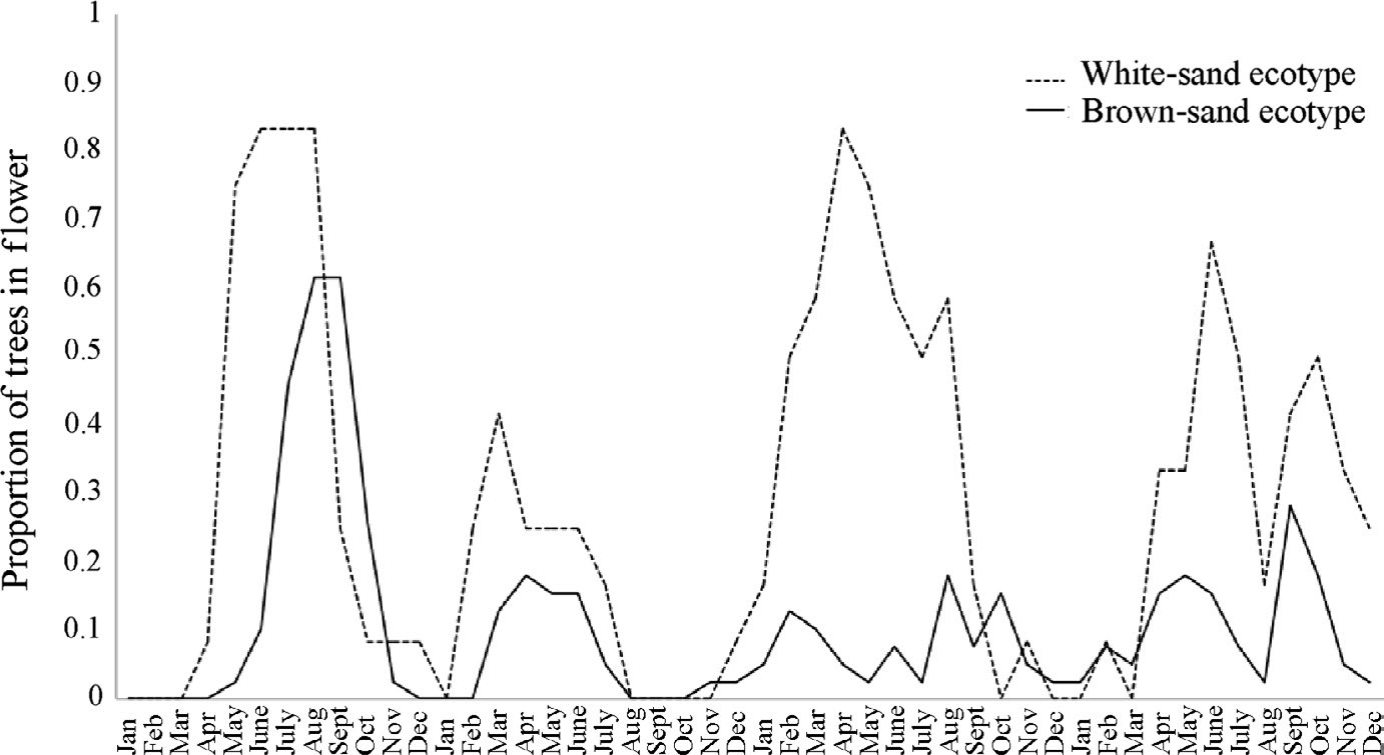
Proportion of white‐sand and brown‐sand individuals of *P. subserratum* in flower across time for January 2006–December 2009

We found little difference in the flowering times of the two ecotypes; however, the abundance of flowering white‐sand and brown‐sand ecotypes varied across the study period with a higher proportion of brown‐sand ecotypes in flower as compared to the white‐sand ecotype (Figure 3). As a result, RI_phenology_ for brown‐sand ecotypes was calculated as 0.69, moderately strong, while RI_phenology_ for white‐sand ecotypes was calculated as −0.23 (Table 1), suggesting that the high abundance of flowering brown‐sand ecotype may facilitate pollen transfer from brown‐sand to white‐sand ecotype trees.

#### 3.1.3 Pollinator assemblage

A total of 14 different morphospecies of insect visitors were observed visiting *P. subserratum* ecotypes. All but three of the insect visitors were identified as stingless bees (Hymenoptera: Apidae: *Meliponini*). Only four morphospecies were observed visiting both ecotypes, and of those, four stingless bee morphospecies (morphospecies A, C and I) were biased toward one ecotype (Table 3). Pollinator assemblages visiting brown‐sand and white‐sand ecotypes differed significantly from one another (*U* = 49, *Z* = −2.61, *p* < .01; Bray–curtis dissimilarity index = 0.82). RI_pollinator overlap_ was calculated as 0.82 for both ecotypes (Table 1).

**TABLE 3:** Total number of pollinator visits observed at white‐sand and brown‐sand ecotypes of *P. subserratum*

Insect visitor	# Visits	
	White‐sand	Brown‐sand
Bee Morph A	22	4
Bee Morph B	6	4
Bee Morph C	9	84
Bee Morph D	0	2
Bee Morph E	0	9
Bee Morph F	0	10
Bee Morph G	0	7
Bee Morph H	0	1
Bee Morph I	2	34
Bee Morph J	0	10
Bee Morph K	0	2
Green fly	0	8
Brown wasp	0	1
Black wasp	0	1
Lepidoptera	1	0

### 3.2 Postpollination isolating barriers

#### 3.2.1 Pollen adherence, germination, and pollen tube growth

**FIGURE 4:**
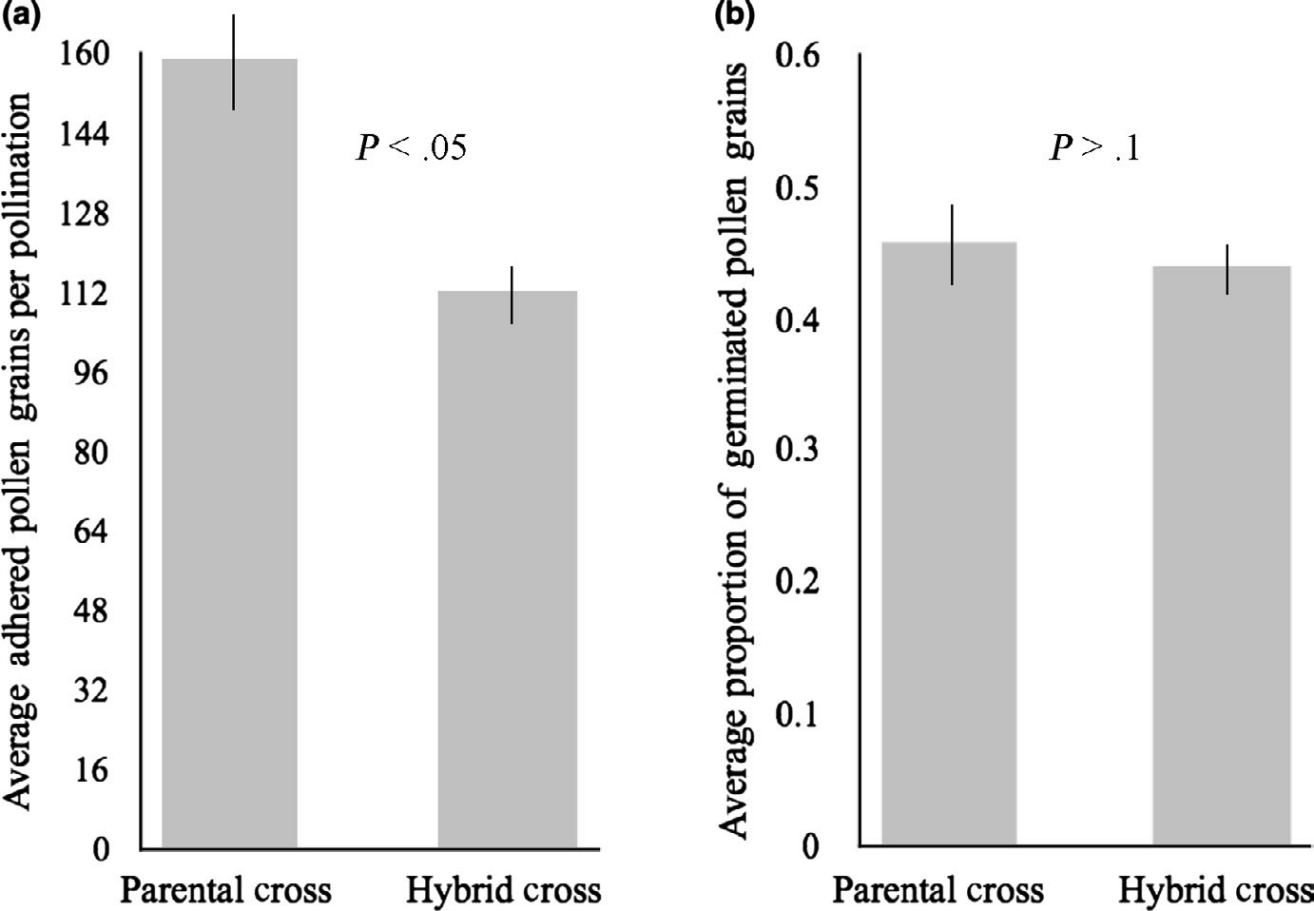
(a) Average number of adhered pollen grains per pollination for parental and hybrid hand crosses using brown‐sand maternal trees. Error bars indicate standard error. (b) Average proportion of adhered pollen grains that germinated pollen tubes in parental and hybrid hand crosses using brown‐sand maternal trees. Error bars are one standard error

Significantly more pollen grains were adhered to stigmas from the parental cross treatment (mean = 159 *SE* ± 16.28) than the hybrid cross treatment (mean = 112 *SE* ± 10.61; *U* = 371, *Z* = 2.36, *p* < .05); however, the proportion of adhered pollen grains which germinated did not significantly differ between treatments with an average of 46% of pollen tubes germinating for parental crosses (*SE* ± 0.03) and an average of 44% of pollen tubes germinating for hybrid crosses (*SE* ± 0.02; *U* = 538, *Z* = −0.27, *p* > .1). None of the negative controls set seed. RI_pollen adhesion_ was 0.18, and RI_pollen germination_ was 0.02 (Table 1, Figure 4).

#### 3.2.2 Fertilization and seed set

**FIGURE 5:**
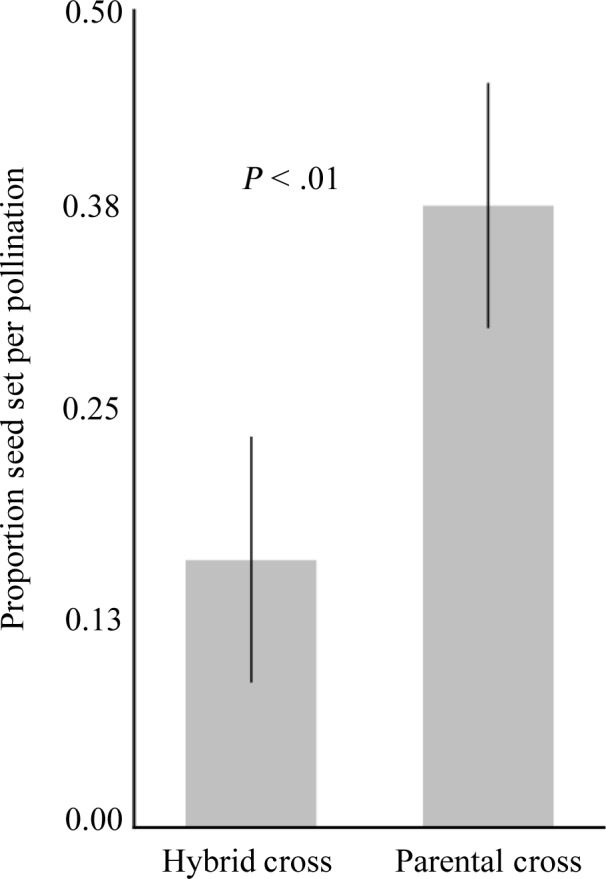
Proportion of hybrid (brown‐sand maternal) and parental (brown‐sand) hand crosses with developing seeds 14 days after pollination. Error bars are one standard error

Seed set per pollination was significantly lower in the hybrid pollination treatment (16.4%) compared to the parental pollination treatment (39%; *p* < .01). RI_seed development_ was 0.40 (Table 1, Figure 5). Seed set for in the positive control flowers was 14%.

### 3.3 Hybrid fitness

#### 3.3.1 Genetic variation, Hardy–Weinberg equilibrium, null alleles, and linkage disequilibrium

We found 7–20 alleles per locus (summed across both populations), and averages across all loci ranged from 4.9 to 6.6 alleles per population. Observed and expected heterozygosity varied from 0.45 to 0.49 and 0.44 to 0.52 across both populations. Inbreeding coefficients were low for both populations (*F*_IS_ = −0.02; 0.07; Table 4). Deviation from HWE was observed in 2 loci (locus prot13 in the white‐sand population and locus prot104 in the brown‐sand population). LD was observed between loci prot70 and prot83, prot29 and prot100, prot70 and prot100, prot83 and prot100, and prot104 and prot100 in the white‐sand population and between loci prot29 and prot67, and prot70 and prot78 in the brown‐sand population.

**TABLE 4:** Ecotypes sampled, number of individuals sampled (*N*), observed heterozygosity (*H*_o_), expected heterozygosity (*H*_e_), average number of alleles (*A*), and inbreeding coefficient (*F*_IS_)

Ecotype	*N*	*H*_o_	*H*_e_	*A*	*F*_IS_
White‐sand	121	0.45	0.44	4.92	−0.02
Brown‐Sand	88	0.49	0.52	6.62	0.07

#### 3.3.2 Ecotypic differentiation and hybrid assignment

**FIGURE 6:**
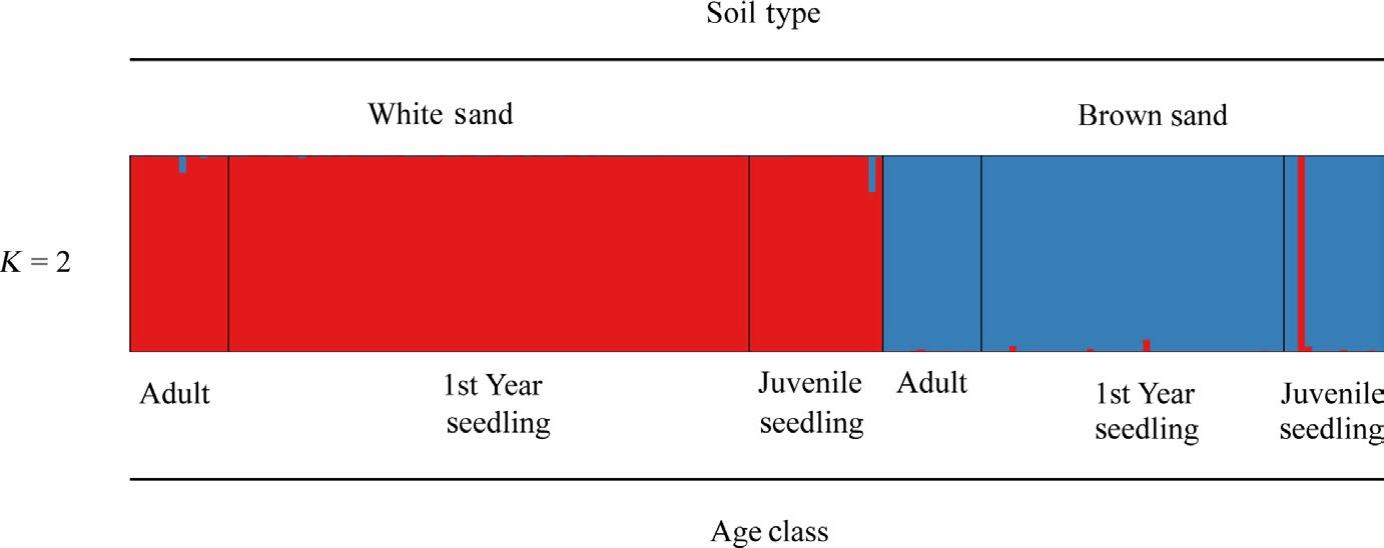
Evolutionary cluster (*K* = 2) inferred from STRUCTURE analysis of 178 white‐sand and brown‐sand ecotypes of *P. subserratum* from three different age classes (adult, first‐year seedling, and juvenile seedling). All seedlings were collected from the seed shadow of maternal trees at a contact zone. Each color represents an inferred character, and each individual is represented by a vertical line shaded according to its probability of assignment to a given population

Pairwise *F*_ST_ values revealed strong genetic differentiation across soil type and little difference across age classes. Values differed only slightly when calculated with and without null allele corrections (Table 5). Population structure analysis also revealed strong patterns of genetic differentiation across soil types. *K* = 2 was the best supported model using evaluation methods of both Evanno, Regnaut, and Goudet (2005) and Pritchard et al. (2000) with the two genetic clusters clearly segregated by soil type (Figure 6). Hybrid assignment analysis confidently assigned all individuals as pure parental white‐sand or pure parental brown‐sand with a posterior probability (pp) > 0.95. One juvenile seedling collected in the seed shadow of a brown‐sand maternal tree was identified as a pure parental white‐sand individual and is likely the result of seed dispersal (Figure 6). No individuals in any age class were identified as *F*_1_, *F*_2,_ or backcross hybrids, and thus, barriers to reproduction appear to be complete prior to seedling establishment and ecologically based low hybrid fitness after seedling establishment is unlikely to be an important barrier to gene flow among brown‐sand and white‐sand ecotypes.

**TABLE 5:** Pairwise *F*_ST_ values for all population pairs

Pop.	WS adult	WS seedling	WS Juvenile	BS adult	BS seedling	BS juvenile
WS Adult	0	0.00	0.00	0.38	0.42	0.37
WS Seedling	0.00	0	0.01	0.43	0.46	0.43
WS Juvenile	0.00	0.01	0	0.40	0.44	0.40
BS Adult	0.37	0.42	0.38	0	0.05	0.01
BS Seedling	0.41	0.46	0.42	0.05	0	0.01
BS Juvenile	0.37	0.43	0.38	0.01	0.01	0

Note

Values below the diagonal are estimated without using corrections for null alleles. Values above the diagonal are estimated using corrections for null alleles.

### 3.4 Total isolation

**FIGURE 7:**
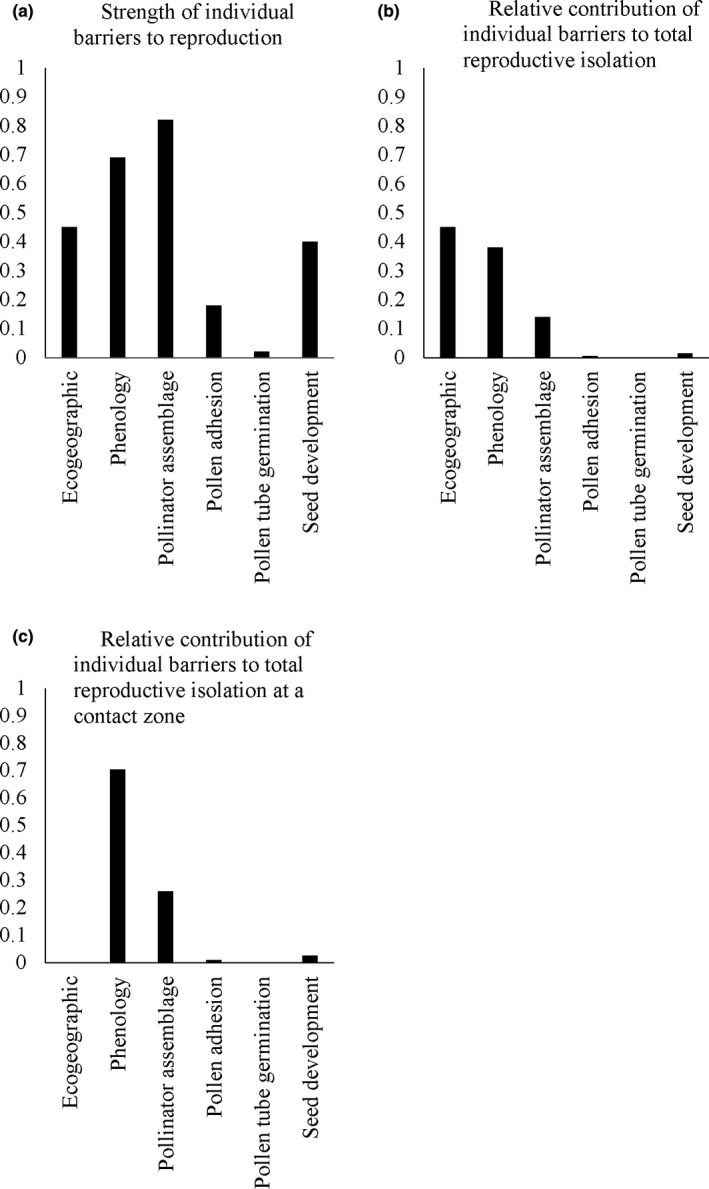
(a) Strength of each individual barrier to reproduction for the brown‐sand ecotype of *P. subserratum*. (b) Relative contribution of each barrier to reproduction to total reproductive isolation for the brown‐sand ecotype. (c) Relative contribution of each barrier to reproduction to total reproductive isolation for individuals found at a contact zone for the brown‐sand ecotype

**FIGURE 8:**
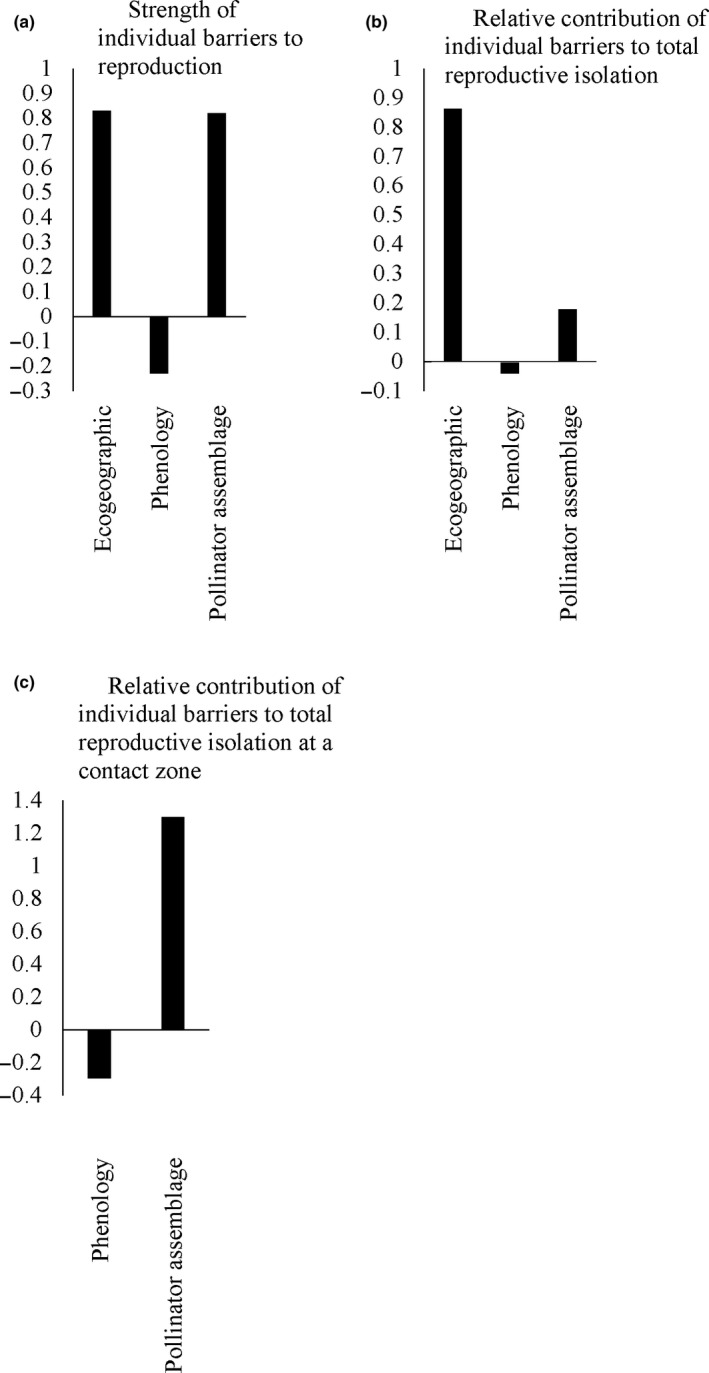
(a) Strength of each individual barrier to reproduction for the white‐sand ecotype of *P. subserratum*. (b) Relative contribution of each barrier to reproduction to total reproductive isolation for the white‐sand ecotype. (c) Relative contribution of each barrier to reproduction to total reproductive isolation for individuals found at a contact zone for the white‐sand ecotype

The strength of each individual barrier to reproduction, the relative contribution of each barrier to total isolation, and the relative contribution of each barrier to isolation at the contact zone (assuming no geographic isolation) for the both ecotypes are summarized in Figure 7. Brown‐sand ecotypes were found to be completely reproductively isolated (RI_Total_ = 0.99) with the majority of the isolation occurring prior to fertilization. Reproductive isolation in sympatry for brown‐sand was also near complete with RI_total_sympatry_ calculated at 0.98. Total reproductive isolation for white‐sand individuals was also near complete even though postpollination barriers could not be accounted for (Figure 8, RI_total_ = 0.96). Reproductive isolation in sympatry for white‐sand ecotypes was also high (Figure 8, RI_total_ = 0.77).

## 4 DISCUSSION

Our study suggests that reproductive isolation at a contact zone between parapatric populations of edaphic specialist white‐sand and brown‐sand ecotypes of *P. subserratum* is almost complete with early‐acting ecological barriers contributing the most to reproductive isolation. We quantified seven potential isolating barriers and demonstrated that ecogeographic isolation accounts for the majority of observed isolation between ecotypes. Both ecotypes overlap almost completely in flowering times; however, differences in pollinator assemblages visiting each ecotype may play an important role in reducing gene flow particularly near contact zones. Later‐acting intrinsic postpollination barriers including reduced heterospecific pollen adhesion and low rates of hybrid fertilization and/or low hybrid seed development were also likely to be important in isolating the two ecotypes. While population genetic analysis by Misiewicz and Fine (2014) detected four individuals identified as *F*_2_ hybrids between white‐sand and brown‐sand populations, the absence of any hybrids, at any age class at the contact zone in this study, suggests that barriers to reproduction are near complete by the time first‐year seedlings are established.

While it is not possible to discern which reproductive barriers were the most important at the initial divergence of edaphic specialist lineages, our findings emphasize the importance of prepollination ecological barriers in maintaining distinct populations of soil specialist trees. These results are consistent with a significant body of literature demonstrating that prepollination, prezygotic barriers to reproduction are stronger than post zygotic barriers (Baack et al., 2015; Kay, 2006; Lowry, Rockwood, et al., 2008) and that multiple barriers are important in completing reproductive isolation (Widmer et al., 2009).

### 4.1 Ecogeographic isolation

The term ecogeographic isolation is used to describe differences in geographic ranges that are dictated largely by intrinsic habitat specialization. Thus, the geographic ranges of differentially adapted taxa should be restricted to their associated habitats (Ramsey et al., 2003; Sobel et al., 2010). We found very high levels of ecogeographic isolation between *P. subserratum* brown‐sand and white‐sand ecotypes, a pattern consistent with other studies (Glennon et al., 2012; Ramsey et al., 2003; Sobel, 2014).

Previous research has demonstrated that there have likely been multiple divergences onto different soil habitats within *P. subserratum* (Misiewicz & Fine, 2014) and that herbivore pressure may be interacting to strengthen selection across habitat boundaries thus contributing to fine‐scale habitat specialization (Fine, Metz, et al., 2013). The strong edaphic associations seen in closely related ecotypes of *P. subserratum* are a pattern shared by numerous tropical tree congeners found across multiple plant families (Fine, García‐Villacorta, Pitman, Mesones, & Kembel, 2010) suggesting that some amount of ecological isolation is likely to be an important factor in diversification and maintenance of diversity in many Amazonian tree lineages.

### 4.2 Flowering phenology

The Lord Howe Palms represent one of the few systems in which barriers to reproductive isolation across soil specialist tree species have been investigated. In this case, studies suggest that shifts to earlier flowering in *Howea forsteriana* as physiological response to nutrient poor calcerarenite soils to which it is adapted are an important barrier to reproduction with *Howea belmoreana (*Hipperson et al., 2016; Savolainen et al., 2006). Contrary to these results, we found little difference in flowering phenology associated with *P. subserratum* growing on different soils. Instead we found that the proportion of individuals flowering was significantly greater on more nutrient rich brown‐sand soils as compared to white‐sand soils thus reducing the probability of gene flow from white‐sand to brown‐sand but increasing the probability of gene flow from brown‐sand trees to white‐sand trees.

### 4.3 Pollinator assemblage

Many studies of reproductive isolation have focused on closely related lineages that show high levels of floral differentiation. Differences in floral architecture and reward can lead to divergence in floral visitors as well as pollinator efficiency resulting in near complete prepollination isolation in sympatry (Karrenberg et al., 2019; Kay, 2006; Ramsey et al., 2003; Scopece, Croce, Lexer, & Cozzolino, 2013; Whitehead & Peakall, 2014). While we detected significant differences in bee communities visiting white‐sand and brown‐sand ecotypes of *P. subserratum*, our system differs from previous species comparisons with similar results in that we observe no difference in floral architecture or color—making this result quite unexpected. Instead *P. subserratum* ecotypes exhibit a generalist floral morphology and display only slight floral variation defined by pubescence on the adaxial surface of the petals of the white‐sand ecotype.

There are a number of explanations for why different pollinator assemblages could be visiting white‐sand and brown‐sand ecotypes in our study. Differences in pollinator visitation could be driven by traits other than floral architecture or color that vary between ecotypes. For instance, white‐sand and brown‐sand ecotypes of *P. subserratum* show distinct differences in their leaf chemistry (Fine, Metz, et al., 2013; Lokvam, Metz, Takeoka, Nguyen, & Fine, 2015). While these chemical differences have been attributed to herbivore defense, they may also be expressed in floral tissue or nectar (Marquis et al., 2016; Stevenson, Nicolson, & Wright, 2017) and some studies have demonstrated that differences in antiherbivore defense chemistry may also indirectly influence bee preference (Kessler, Halitschke, & Poveda, 2011; Adler, Seifert, Wink, & Morse, 2012 and reviewed in Stevenson et al., 2017). Alternatively, variation in pollinator visitation may be the result of pollinator habitat preference driven by differences in microclimate, food sources, or nesting resources in white‐sand and brown‐sand habitats. For instance, Misiewicz, Kraichak, & Rasmussen (2014) demonstrated fine‐scale turnover in stingless bee communities in different soil habitats over distances of <1 km. If pollinators are foraging over very small distances or they themselves are habitat specialists restricted to forests found on different soil types, then turnover in pollinator communities visiting white‐sand and brown‐sand trees may be the indirect result of pollinator habitat preference and little to do with floral preference.

Finally, while these results provide intriguing preliminary data, longer and more frequent observations on a greater number of trees will be necessary to confirm the extent to which floral visitors differ among these ecotypes and the mechanisms that may be driving partitioning of pollinator communities.

### 4.4 Pollen adhesion and germination

Intraspecific pollen–pistil recognition at the stigma surface is a common mechanism in plants to avoid conspecific pollen germination and growth that would deplete female tissue resources (Heizmann, Luu, & Dumas, 2000; Howard, 1999). If pollen transfer between populations that are locally adapted to different habitats results in less fit hybrid offspring, then natural selection should favor individuals with traits that limit the production of costly hybrid offspring (Dobzhansky, 1937, 1940). Alternatively, Searcy and MacNair (1990) demonstrated that adaptation across an edaphic gradient was associated with selection against the alternative ecotype in the pistil suggesting that in some cases, ecological divergence may directly lead to the formation of intrinsic prezygotic barriers to reproduction.

We found significantly lower levels of white‐sand pollen adhered to brown‐sand flowers than we did brown‐sand pollen adhered to brown‐sand flowers but no difference in the proportion of adhered pollen germination between treatments. Our results suggest that pollen–pistil incompatibilities limiting pollen adhesion could be an early‐acting postpollination barrier to reproduction. However, we cannot exclude alternative explanations for these results. Lower levels of pollen adherence in hybrid hand pollinations could also be explained if white‐sand flowers produce lower overall quantities of pollen than brown‐sand ecotype flowers. Although dehiscent anthers of both white‐sand and brown‐sand ecotypes were observed using a dissecting microscope and flowers of both ecotypes appeared to release equally large quantities of pollen, we did not quantify pollen production. Regardless, pollen adhesion in hybrid crosses, while lower than in parental crosses, was still high (averaging 112 pollen grains per stigma) and the proportion of adhered grains that germinated did not differ between treatments (~45%). Given that each flower only contains two ovules, hybrid crosses still had many more adhered and germinated pollen grains than would be required for successful seed set suggesting that if initial pollen–pistil incompatibilities are present, they are weak and far from complete.

### 4.5 Fertilization/seed development

We detected additional genetically based barriers to reproduction in hand crossing experiments. However, because we were unable to visualize pollen tube growth in the style for any treatment, we could not discern the ultimate cause for reduced hybrid success. Failure of hybrid crosses to produce seeds could be the result of the lack of fertilization through genetically based prezygotic barriers such as pollen tube/style incompatibilities—an important barrier to reproduction in neotropical *Costus* (Yost & Kay, 2009). Alternatively, fertilization may have been successful and post zygotic barriers to reproduction such as genetically based postzygotic hybrid mortality.

Because barriers to reproduction continue to accumulate after reproductive isolation is complete, it is not possible to distinguish the order in which they arose (Rieseberg & Willis, 2007). Scopece, Musacchio, Widmer, and Cozzolino (2007) found that the strength of intrinsic postzygotic barriers is correlated with genetic distance among orchids and therefore may evolve later in the speciation process; however, this pattern does not necessarily hold across other plant lineages (Moyle, Olson, & Tiffin, 2004). For instance, Bomblies et al. (2007) demonstrated that epistatic interactions among loci can lead to hybrid necrosis in intraspecific crosses of *Arabidopsis thaliana*. Two studies examining barriers to reproductive isolation in woody plants found that postzygotic barriers may evolve before prezygotic barriers (Johnson et al., 2015; Stacy et al., 2017). For example, Johnson et al. (2015) found that sympatric *Cyrtandra* species on the Hawaiian Islands are isolated primarily by postzygotic barriers to reproduction that likely arose in allopatry. Regardless of the point in which intrinsic postzygotic barriers to reproduction arise, it is generally agreed upon that while they typically contribute less to total reproductive isolation than early‐acting prepollination barriers, they often play a particularly important role in maintaining isolation during periods of environmental variability when prepollination barriers may be weakened (Widmer, Lexer & Cozzolino., 2009).

## 5 CONCLUSIONS

We were able to identify five active barriers to reproduction between soil specialist ecotypes of the Amazonian tree, *P. subserratum*. Although other studies have explored the relative strength of prepollination versus postpollination and prezygotic versus postzygotic barriers to reproduction in plants, most of these studies have focused on temperate systems and none have comprehensively explored barriers in tropical trees. Our study was limited due to our inability to safely access flowers in the canopy from a large number of trees. As a result, the number of maternal trees used to for hand cross‐pollination experiments was low, and we were not able to compare the effectiveness of reproductive isolating barriers in both directions. The absence of hybrid seedlings in the seed shadows of white‐sand and brown‐sand maternal trees at contact zones suggests that total reproductive isolation is nearly complete in both directions. The results presented here serve as a foundation for further investigation.

While many studies have found that prezygotic barriers to reproduction are stronger than postzygotic barriers because they are early acting, oftentimes it is not possible to determine which barriers evolved first and therefore which barriers were most important at the point of initial divergence. While intrinsic postzygotic isolation in plants can evolve rapidly, it is often correlated with genetic divergence (Widmer et al., 2009). If *P. subserratum* ecotypes evolved in allopatry, postzygotic barriers could have evolved first and prezygotic barriers observed in this study may be the result of reinforcement. Reinforcement is thought to occur when strong selection to avoid wasting costly gametes in the production of unfit hybrids leads to the evolution of strong prezygotic reproductive barriers in sympatric populations upon secondary contact thus completing the speciation process (Dobzhansky, 1937). Reinforcement in plants is commonly associated with shifts in floral morphology leading to the partitioning of pollinators and the evolution of pollen–stigma incompatibilities. While the latter may be at play in our system, there are very few differences in floral morphology between the two ecotypes that explain the observed differences in visiting pollinator communities. Alternatively, early‐acting prezygotic barriers could have evolved first followed by development of postzygotic barriers. Previous studies of white‐sand and nonwhite‐sand ecotypes of *P. subserratum* have found that they are attacked by different assemblages of herbivores, exhibit quantitative and qualitative differences in antiherbivore defense compounds, and that different soil ecotypes utilize different growth strategies. Together, these results suggest that herbivore pressure may be a particularly important factor in driving local adaptation across edaphic boundaries. If secondary compounds that deter herbivores are expressed in the floral structures, nectar, or pollen, they could simultaneously attract or deter pollinators (Adler, 2000; Kessler & Halitschke, 2009; Raguso, 2008) potentially leading to the partitioning of pollinator communities among diverging populations (Marquis et al., 2016). Additionally, some work has suggested that certain herbivore defense chemicals may be correlated with pollen–stigma interactions potentially influencing the success of intraspecific pollen germination and pollen tube growth (Rejón et al., 2013). In this case, divergence in secondary chemistry could lead directly to prezygotic reproductive isolation.

Regardless of the order in which these barriers may have evolved, our results suggest that multiple pre‐ and postzygotic barriers to reproduction are maintaining reproductive isolation between edaphically divergent populations of the tropical tree, *P. subserratum*. This study provides the first comprehensive evaluation of reproductive isolating barriers in an Amazonian tree and contributes to our basic knowledge of plant speciation.

## CONFLICT OF INTEREST

The authors declare no conflict of interest.

## AUTHOR CONTRIBUTION

**Tracy M. Misiewicz:** Conceptualization (lead); Data curation (lead); Formal analysis (lead); Investigation (lead); Methodology (lead); Project administration (lead); Writing‐original draft (lead); Writing‐review & editing (lead). **Tracey S. Simmons:** Formal analysis (supporting); Methodology (supporting); Writing‐original draft (supporting); Writing‐review & editing (supporting). **Paul V. A. Fine:** Conceptualization (supporting); Funding acquisition (lead); Resources (lead); Writing‐original draft (supporting); Writing‐review & editing (supporting).

## Supporting information

Table S1

## Data Availability

Data availability: Tree coordinates; hand‐cross data, pollen/pollen tube data, experimental tree summary data, and microsatellite data have been submitted DataDryad: https://doi.org/10.6078/D12X1K.
