# Supplementary material for: The contribution of multiple barriers to reproduction between edaphically divergent lineages in the Amazonian tree Protium subserratum (Burseraceae)
Source: Ecol Evol. 2020 Jun 17;10(13):6646–63. doi: 10.1002/ece3.6396 (PMC7381562; doi:10.1002/ece3.6396)
Supplement: Supplementary file 1 — Table S1 [file ECE3-10-6646-s001.pdf]

Supplementary 1. ID, Ecotype, Coordinates and Sex for Experimental Trees

Tree ID	Ecotype	UTM 18M		Sex	Pollinator assemblage	Pollen adhesion and germination	Fertilization/seed development	Maternal Tree hybrid fitness
AM-Blanco-C	White-sand	674225	9560320	Male		X	X	
AM-Blanco-D	White-sand	674242	9560475	Male	X			
AM-Blanco-E	White-sand	674232	9560510	Female	X			
AM-Blanco-F	White-sand	674230	9560520	Male	X		X	
AM-Blanco H	White-sand	674257	9560498	Male	X		X	
AM-Blanco-M	White-sand	674327	9560384	Female				X
AM-Blanco-T	White-sand	674262	9560350	Female				X
AM-Blanco-U	White-sand	0674368	9560027	Female				X
AM-Hib-A	Brown-sand	674368	9560265	Female				X

Supplementary 1. ID, Ecotype, Coordinates and Sex for Experimental Trees

AM-Hib-C	Brown-sand	674364	9560292	Male			X	
AM-Hib-D	Brown-sand	674362	9560293	Female			X	X
AM-Hib-L	Brown-sand	674645	9560210	Male	X	X	X	
AM-Hib-N	Brown-sand	674394	9560314	Female				X
AM-Hib-O	Brown-sand	674202	9560448	Female	X	X	X	X
